# Are Corsets a Necessity

**Published:** 1888-09

**Authors:** Annie Jenness Miller


### ARE CORSETS A NECESSITY?

The perfectly shaped woman is a very ideal of refined curves; and the fact that women lose their graceful proportions with the passing years, is not due, as is charged, to maternity and age, but to false living, dressing and inattention to the preservation of the figure instead. To keep one's figure, care must be taken, and that not the care of buying the stiffest and most unyielding corsets possible, with which to confine the body, but the care which preserves inherent grace: the care which keeps down the adipose tissue; the care which prevents the bosom from becoming heavy and gross; the care which keeps the muscles flexible and well poised; in other words, the care which insures the gradual and desirable change from the slight, girlish and undeveloped proportions of youth, into the symmetrical, well rounded and luxurious curves of the matron.

This care becomes a fine art with the few women who give it thought but the art is legitimate and commendable. We will not deny that the woman who throws aside her corsets and reposes with hands folded to grow *fat* will have a sufficiently unshapely and unattractive form to call forth a protest; and so, too, will the woman of tremendous nervous energy who reduces herself to a skeleton, fail to attract the eye which delights in firm, fair flesh; but *the women who use plenty of cold water and friction on the body, who take rational physical exercise, and employ scientific means for development where they have it not, who hold the chest firmly raised and breathe properly, will have decided natural advantages over the foolish ones who depend upon the corset for form.*

We do not doubt that many women wear corsets who do not lace them unnaturally, and with the present style of close-fitting bodice they look the better for it: but the tendency once within the corset is to pull upon the lacings until the natural organs of support lose their power and yield place to the artificial ; and even where there is no deliberate tightening of the corset-string there is a tacit understanding that a few added pounds of flesh shall not be considered reason sufficient for "letting out," and thus rigidity soon reigns and grace becomes impossible.

These are the reasons why those of us who believe in health and freedom for the body say, eschew the corset altogether. Were women possessed of the artistic sensibility which would insure the triumph of true proportion, they might then wear a well-shaped waist, or if the name suits better, corset, without disadvantage to health or figure ; but the moment that the corset idea is admitted abuse follows, and before long disproportion reigns and physical weakness becomes general. Once exaggeration begins, the bosom goes up higher and higher, the waist line comes down, throwing the hips out, the shoulders are squared, and the elbows take an angle ; in fact, the whole *tout ensemble* becomes one of stiffness, awkwardness and disproportion. Stylish ? yes ; beautiful ? no.

Were corsets worn to meet the requirements of nature, instead of compelling nature to meet the shapes and purposes of the corset, there would be no cause for quarrel between us.—*Annie Jenness Miller.*

